# High-Load Resistance Exercise Augments Androgen Receptor–DNA Binding and Wnt/β-Catenin Signaling without Increases in Serum/Muscle Androgens or Androgen Receptor Content

**DOI:** 10.3390/nu12123829

**Published:** 2020-12-15

**Authors:** Thomas D. Cardaci, Steven B. Machek, Dylan T. Wilburn, Jeffery L. Heileson, Darryn S. Willoughby

**Affiliations:** 1 Department of Health, Human Performance and Recreation, Robbins College of Health and Human Sciences, Baylor University, Waco, TX 76706, USA; tcardaci@email.sc.edu (T.D.C.); steven_machek2@baylor.edu (S.B.M.); dylan_wilburn1@baylor.edu (D.T.W.); jeffery_heileson@baylor.edu (J.L.H.); 2 Department of Exercise Science, Arnold School of Public Health, University of South Carolina, Columbia, SC 29208, USA; 3 School of Exercise and Sport Science, Mayborn College of Health Sciences, University of Mary Hardin-Baylor, Belton, TX 76513, USA

**Keywords:** androgen receptor, β-catenin, skeletal muscle, cell signaling, Wnt signaling, hypertrophy, resistance exercise, testosterone, dihydrotestosterone, load

## Abstract

The purpose of this study was (1) to determine the effect of single bouts of volume- and intensity-equated low- (LL) and high-load (HL) full-body resistance exercise (RE) on AR-DNA binding, serum/muscle testosterone and dihydrotestosterone, muscle androgen receptor (AR), and AR-DNA binding; and, (2) to determine the effect of RE on sarcoplasmic and nucleoplasmic β-catenin concentrations in order to determine their impact on mediating AR-DNA binding in the absence/presence of serum/muscle androgen and AR protein. In a cross-over design, 10 resistance-trained males completed volume- and intensity-equated LL and HL full-body RE. Blood and muscle samples were collected at pre-, 3 h-, and 24 h post-exercise. Separate 2 × 3 factorial analyses of variance (ANOVAs) with repeated measures and pairwise comparisons with a Bonferroni adjustment were used to analyze the main effects. No significant differences were observed in muscle AR, testosterone, dihydrotestosterone, or serum total testosterone in either condition (*p* > 0.05). Serum-free testosterone was significantly decreased 3 h post-exercise and remained significantly less than baseline 24 h post-exercise in both conditions (*p* < 0.05). In response to HL, AR-DNA binding significantly increased at 3 h post-exercise (*p* < 0.05), whereas no significant differences were observed at any time in response to LL (*p* > 0.05). Moreover, sarcoplasmic β-catenin was significantly greater in HL (*p* < 0.05) without significant changes in nucleoplasmic β-catenin (*p* > 0.05). In conclusion, increases in AR-DNA binding in response to HL RE indicate AR signaling may be load-dependent. Furthermore, despite the lack of increase in serum and muscle androgens or AR content following HL RE, elevations in AR-DNA binding with elevated sarcoplasmic β-catenin suggests β-catenin may be facilitating this response.

## 1. Introduction

Androgens, such as testosterone and dihydrotestosterone (DHT), play a pivotal role in muscle specific gene and protein expression, which can ultimately lead to skeletal muscle hypertrophy [1]. Primarily, androgens exert their anabolic effects through the bloodstream where they interact with androgen receptors (AR) in skeletal muscle. Specifically, free/unbound testosterone diffuses across the sarcolemma in skeletal muscle, where a portion is converted to the more biologically active DHT (relative greater AR binding affinity and reduced disassociation rate) by the enzyme, 5α-reductase [2,3]. Once bound by the androgen, the activated AR undergoes a conformational change causing a dissociation from the heterocomplex of heat shock proteins and other co-chaperones, ultimately resulting in dimerization. After dissociation, it is then considered an active AR complex and is translocated into the nucleus where it binds to the androgen response element (ARE) upstream of AR gene promoter regions. The AR gene, the p21 cyclin-dependent kinase inhibitor protein 1 gene, and the insulin-like growth factor-1 gene have all been found to contain AREs [4]. This AR-DNA binding results in up-regulation of these genes (and others) which play significant roles in skeletal muscle anabolism and contribute to hypertrophy of the muscle [5].

It is thought that the acute increase in serum/muscle androgen and/or AR content are responsible for the up-regulation in AR signaling. While this has been reported [4,6,7,8,9,10,11,12,13,14,15,16,17], AR activation and subsequent DNA-binding has been shown to increase in response to resistance exercise (RE) independent of an androgenic response as well [18,19,20,21,22,23,24]. Additionally, acute AR mRNA and/or protein expression in response to RE has been shown to increase [17,18,25], decrease or not change in a number of studies [6,13,23,25,26,27,28]. This suggests that increases in AR signaling may not be primarily dependent on rises in androgens or AR protein concentrations. However, just as relevant, the variety of methodological approaches between RE protocols may be influencing the differential results.

Recent research suggests increases in AR signaling in response to mechanical loading may be due to up-regulation in specific AR-interacting proteins [23,29,30]. AR-interacting proteins may increase AR signaling by modulating AR binding affinity and/or AR activation via ligand-dependent or -independent processes [31,32]. Specifically, β-catenin, an important multifunctional protein involved in wingless-type mouse mammary tumor virus integration site (Wnt) signaling, has been shown to be a transcriptional co-activator of the AR [33,34,35]. Mechanistically, β-catenin concentrations increase within the sarcoplasm in response to a series of regulatory steps. This process involves: (1) the binding of a Wnt ligand to the extracellular cysteine rich domain on the transmembrane Frizzled receptor, (2) phosphorylation/activation of the protein disheveled by low-density lipoprotein receptor-related protein (LDLR-LRP) -5 or -6 co-receptors, (3) blocking of glycogen synthase kinase-3β activity (GSK-3β) by sequestering GSK-3β via the inhibitory protein frequently rearranged in advanced T cell lymphomas (Frat), and (4) inactivation of the “destruction complex” resulting in decreased phosphorylation and down-regulating proteasomal degradation of β-catenin [36,37,38,39]. This leads to armadillo repeats 1–6 of β-catenin to interact with the activation function-2 (AF-2) region (within the ligand-binding domain (LBD)) to facilitate LBD-NTD interactions and the ensuing formation of a “ligand-binding pocket” and/or stabilization of the bound androgen [32,34,39,40,41]. Following β-catenin-mediated activation, the AR complex translocates into the nucleus where it binds to the ARE on the target gene up-regulating muscle specific gene expression, which in turn can play an important regulatory role in skeletal muscle growth. Currently, there are extremely limited data investigating β-catenin-AR interactions in RE models. Thus far, the existing data suggest that β-catenin-mediated AR signaling does appear to be responsive to RE [23,42]. However, β-catenin’s response to various RE design variables and the specific mechanisms remain largely unexplored.

It is generally thought that higher volume and intensity RE are needed to elicit an appropriate stimulus for increasing AR signaling activity. While this may be the case, RE load may be the key variable responsible for increasing AR activity, provided it is accompanied with appropriate training volume and intensity. This is due to the overwhelming role load has on recruitment of motor units and the subsequent fiber type-specific metabolic and/or contractile stress placed on skeletal muscle. However, research investigating the load’s impact on muscular adaptations is commonly misinterpreted due to suboptimal methodological approaches and inconsistencies in terminology. For example, much of the literature does not clearly define and differentiate between intensity and load. In RE, intensity is commonly defined as a percent of 1 repetition maximum (1 RM) [43]. However, it is more appropriate to define this as “load”. Intensity may be more appropriately defined as the number of repetitions performed at a given RM [44]. For example, performing 10 repetitions at a 10 RM (which is approximately 75% of 1 RM) until volitional failure would be considered an intensity of 100%. These semantic differences and lack of clearly defined variables have contributed to some of the confusion in this area. Furthermore, rarely is the effect of load on skeletal muscle adaptations examined without being affected by confounding variables such as volume or intensity. This is problematic since volume and intensity have both been shown to be important factors influencing androgenic hormone responses following RE [45]. Therefore, the purpose of this study was (1) to determine the effect of single bouts of volume- and intensity-equated low (LL) and high-load (HL) full-body RE on AR-DNA binding, serum/muscle testosterone and DHT, AR muscle protein content, and AR-DNA binding and (2) to determine the effect of on sarcoplasmic and nucleoplasmic β-catenin concentrations in order to determine their impact on mediating AR-DNA binding in the absence/presence of serum/muscle androgen and AR protein.

## 2. Materials and Methods

### 2.1. Experimental Approach

Participants visited the laboratory on 3 separate occasions in the following manner: visit 1 = entry/familiarization, medical/physical activity screening, and RE max testing; visit 2 = LL (50% 1 RM) RE; visit 3 = HL (80% 1 RM) RE session. Each visit was 7–10 days following the previous visit and participants were instructed to refrain from exercise for 48 h prior to RE max testing and RE protocols. In a crossover design and volume-equated manner, participants performed identical full-body RE consisting of barbell bench press, angled leg press, lat pulldown, and unilateral leg extension. During LL RE, participants performed 50% of their one repetition maximum to volitional failure for 4 sets in each exercise. Total exercise load volume (sets × repetitions × load) was calculated and equated in the HL RE during visit 3. During HL, sets were performed until participants reached the volume in order to match the total volume performed during LL. This allowed for volume and intensity to be equated between the two conditions. Each session involved the gathering of data for the analysis of biochemical and hormonal markers of blood and muscle metabolite changes.

### 2.2. Participants

Ten apparently healthy, recreationally resistance-trained (regular, consistent resistance training (i.e., thrice weekly) for at least 1 year prior to the onset of the study) men between the ages of 18–35 volunteered for this study. Resistance training status was confirmed by a leg press 1-RM, which was compared to normal strength-to-body weight ratios. Participants were eligible for inclusion if their strength-to-body weight ratio was ≥2.82 times body weight [46]. Participants were also included if they were at low risk for cardiovascular disease with no contraindications to exercise as outlined by the American College of Sports Medicine (ACSM) and had not consumed any nutritional supplements (excluding multi-vitamins) one month prior to the study. All eligible participants signed university-approved informed consent documents and received a written and verbal explanation of the study design. All study procedures were approved by the Institutional Review Board at Baylor University (approval #1521229-3) and conformed to the ethical consideration of the Declaration of Helsinki. Mean (±SD) participant descriptive information (anthropometrics, baseline health assessments, resistance training experience) are found in Table 1.

### 2.3. Resistance Exercise Max Testing

To determine muscular strength and proper RE load prescription, participants performed 1-RM tests for bench press and angled leg press and 10-RM tests for unilateral leg extension and lat pulldown in accordance with the National Strength and Conditioning Association (NSCA) guidelines. Leg press foot placement and bench press/lat pulldown handgrip width were recorded and held constant over all testing conditions in order to maintain consistency. To ensure participants were moving through the full range of motion during each repetition, a goniometer was used to establish 90 degrees of knee flexion on the leg press. Moreover, during the bench press, participants were instructed to touch their chest with the barbell and extend their elbows to full extension to constitute a completed repetition. Similarly, participants were required to fully extend their elbows and pull the cable attachment until it made contact with their sternum/chest during the lat pulldown as well as fully extend their knees to constitute a completed repetition during the unilateral leg extension.

Participants warmed up by completing 5 to 10 repetitions at approximately 50% of the estimated 1 RM/10 RM. Then participants rested for 1 min and then completed 3 to 5 repetitions at approximately 70% of the estimated 1 RM/10 RM. Load was then increased conservatively and the participant attempted to lift the load for 1/10 repetition(s). If the lift was successful, the participant rested for 5 min before attempting the next weight increment. As per NSCA guidelines, load was increased by 2.5–5% for upper body exercises and by 5–10% for lower body exercises. This procedure was continued until the participant failed to complete the lift. The 1 RM/10 RM was recorded as the maximum weight that the participant was able to lift for 1/10 repetition(s).

### 2.4. Resistance Exercise Protocol

During visits 2 and 3 participants performed full-body RE protocols consisting of barbell bench press, angled leg press, lat pulldown, and unilateral leg extension. As our main experimental variable, load varied between LL and HL RE while keeping all other controllable variables constant. During LL RE, participants performed 3 sets of each exercise at 50% 1 RM and were taken to volitional failure following the previously described warm-up protocol. During HL RE, participants performed each exercise at 80% 1 RM to volitional failure. Additionally, volume was equated between the two visits. Due to the greater amount of volume that can be accumulated with a lighter load, additional sets for each exercise were utilized (if necessary) in order to equate volume between LL and HL conditions. Moreover, when fatigue/failure occurred, study personnel provided assistance to help re-rack the weight safely. In all cases, 2–4 min rest occurred between all sets and exercises. Due to the diurnal nature and dietary influence of the biomarkers being investigated, participants reported to the laboratory upon waking and in a fasted state at 08:53 (±0:55) and 08:37 (±1:00) for LL and HL, respectively. Moreover, in order to minimize nutritional mediation of the markers we were investigating, participants received a standardized nutrition bar 30 min prior to RE (Power Bar^®^, Premier Nutrition Corporation, Kings Mountain, NC, USA, (carbohydrate: 25 g, protein: 20 g, fat: 6 g, fiber: 4 g)). Additionally, as auxiliary measures of exercise intensity, muscle soreness and a rating of perceived of exertion (RPE) of each condition was gathered. Muscle soreness was assessed pre-exercise, 3 h post-, and 24 h post-exercise via a visual analogue scale in which participants were instructed to draw an intersecting mark across a 13-cm scale (0 = no soreness, 13 = extreme soreness). The distance of each mark was measured from 0, and the measurement was used as the observed muscle soreness value. RPE was assessed immediately post-exercise via the modified Borg Scale (0–10). Lastly, in an attempt to control for variations in RE performance, skeletal muscle strength, and proper recovery, RE protocols were scheduled within 2 h of each other and separated by 7–10 days [47].

### 2.5. Body Composition, Dietary, and Hydration Analysis

Total body mass (kg) and height (cm) were determined on a standard dual beam balance scale (Detecto Bridgeview, IL, USA). Percent bone mineral content, body fat, fat mass, and lean body mass were determined using dual-energy X-ray absorptiometry (DEXA) (Hologic Discovery Series W, Waltham, MA, USA). Participants were required to record their dietary intake for 48 h prior to RE max testing and protocols. Participants’ diets were not be standardized but were asked to keep their dietary habits consistent throughout the study. The dietary recalls were evaluated with the MyFitnessPal mobile or desktop application (Under Armor Inc., Baltimore, MD, USA) to determine the average daily macronutrient consumption of fat, carbohydrate, and protein in the diet for the duration of the study. Since previous research has demonstrated individuals who are dehydrated will have an attenuated testosterone response with RE [48] and to account for shifts in plasma volume, prior to the max testing and RE protocols, total body water using bioelectrical impedance analysis (Tanita, Tokyo, Japan) and packed cell volume were assessed. 

### 2.6. Muscle Biopsies

Percutaneous muscle biopsies (~30 mg) were obtained from the middle portion of the vastus lateralis muscle of the dominant leg (midpoint between the patella and the greater trochanter of the femur) at a depth between 1 and 2 cm using the fine needle aspiration technique. Muscle tissue was extraction using the TRU-CORE^®^ 1 Automatic Biopsy Instrument (Angiotech, Medical Device Technologies, Inc., Gainsville, FL, USA) after subcutaneous administration of the local anesthetic (1 mL of 1% lidocaine/xylocaine). After the initial biopsy, the following biopsy attempts were made to extract tissue from approximately the same location as the initial biopsy by using the pre-biopsy scar, depth markings on the needle, and a successive incision that was made approximately 0.5 cm to the former from medial to lateral. After removal, adipose tissue was trimmed from the muscle specimens and was immediately frozen and stored at −80 °C for later analysis. Three muscle samples were obtained at visits 2 and 3 for a total of 6 muscle biopsies performed during the course of the study. Biopsies were taken pre-exercise and at 3 and 24 h post-exercise during visits 2 and 3.

### 2.7. Venipuncture

Venous blood samples were obtained into 10 mL vacutainer tubes using a 21-gauge phlebotomy needle inserted into the antecubital vein. Blood samples stood at room temperature for 10 min and then centrifuged. The serum was then removed and frozen at −80 °C for later analysis. Six blood samples were obtained during the course of the study. The blood samples were collected pre-exercise and at 3 and 24 h post-exercise during visits 2 and 3.

### 2.8. Skeletal Muscle Total, Sarcoplasmic, and Nucleoplasmic Protein Extraction

A portion of each muscle sample was weighed and homogenized using a commercial tissue extraction reagent (Invitrogen Corporation, Camarillo, CA, USA) and a tissue homogenizer. Following total protein extraction, total sarcoplasmic and nucleoplasmic proteins were isolated separately using cytoplasmic and nuclear extraction buffers (Aviva Systems Biology Corporation, San Diego, CA, USA). All extracts were supplemented with phenylmethanesulfonyl fluoride and a protease inhibitor cocktail (Sigma Chemical Company, St. Louis, MO, USA) with broad specificity for the inhibition of serine, cysteine, and metallo-proteases. Protein contents for total protein, sarcoplasm-, and nuclear-extracted samples were analyzed in duplicate and determined spectrophotometrically at a wavelength of 750 nm (Bio-Rad Hercules, CA, USA) and using bovine serum albumin as the standard [49].

### 2.9. Serum Total and Free Testosterone Analysis

The concentrations of serum total and free testosterone were assessed via commercially-available enzyme-linked immunosorbent assay (ELISA) kits (Eagle Biosciences Incorporation, Nashua, NH, USA). The specificity of these kits are 100% with the sensitivity estimated to be 0.022 ng/mL and 0.018 pg/mL, respectively, for total and free testosterone. Samples were analyzed in duplicate and absorbances were read at a wavelength of 450 nm. Unknown concentrations were determined by linear regression against known standard curves using commercial software (Microplate Manager, Bio-Rad, Hercules, CA, USA). The overall intra-assay percent coefficients of variation were 2.37% (±2.54) and 2.1% (±1.98) and, respectively, for total and free testosterone.

### 2.10. Intramuscular Testosterone and Dihydrotestosterone (DHT) Analysis

The same ELISA kit employed for serum free testosterone was used to analyze intramuscular testosterone concentrations. Similarly, intramuscular DHT was assessed using a commercially available ELISA kit (Eagle Biosciences Incorporation, Nashua, NH, USA). These assays were performed using the total muscle protein cellular extracts [23,28]. The specificity of the free testosterone and DHT ELISA kits were both 100% with the sensitivity estimated to be 0.018 pg/mL and 6 pg/mL, respectively. All samples were analyzed in duplicate and absorbances were determined at a wavelength of 450 nm using a microplate reader (iMark, Bio-Rad, Hercules, CA, USA) against known standard curves, and final concentrations expressed relative to total protein concentration. The overall intra-assay percent coefficients of variation were 1.31% (±1.15) and 3.35% (±2.71), respectively, for intramuscular testosterone and DHT.

### 2.11. Intramuscular Androgen Receptor Analysis

Total AR protein content was assessed in total muscle protein cellular extracts via commercially available ELISA kits (MyBioSource Incorporation, San Diego, CA, USA). The assay is 100% specific and has a sensitivity of 0.1 ng/mL. All samples were analyzed in duplicate and absorbances were determined at a wavelength of 450 nm using a microplate reader (iMark, Bio-Rad, Hercules, CA, USA) against known standard curves, and final concentrations expressed relative to total protein concentration. The overall intra-assay percent coefficient of variation was 7.91% (±6.64).

### 2.12. Androgen Receptor—DNA Binding Analysis

AR-DNA binding was quantified in nucleoplasmic extracts by a commercially available ELISA kit (Aviva Systems Biology Corporation, San Diego, CA, USA). This particular immunoassay utilizes the qualitative technique of an indirect ELISA and the incorporation specific double-stranded (dsDNA) oligonucleotides (representing the ARE (5′-AGAACA-3′)). The assay is 100% specific and has a sensitivity of 0.3 μg of AR–DNA binding in nuclear-extracted MCF7 cells. All samples were analyzed in duplicate and absorbances were determined at a wavelength of 450 nm using a microplate reader (iMark, Bio-Rad, Hercules, CA, USA). Absorbances were then expressed relative to total nucleoplasmic protein content. The overall intra-assay percent coefficient of variation was 9.89% (±7.59).

### 2.13. Sarcoplasmic and Nucleoplasmic β-Catenin Analysis

Sarcoplasmic and nucleoplasmic β-catenin were assessed via commercially available ELISA kits (Biovision Incorporated, Milpitas, CA, USA) and performed using the sarcoplasmic and nucleoplasmic extracts. The assay is 100% specific and has a sensitivity of 0.156 ng/mL. All samples were analyzed in duplicate and absorbances were determined at a wavelength of 450 nm using a microplate reader (iMark, Bio-Rad, Hercules, CA, USA) against known standard curves, and final concentrations expressed relative to sarcoplasmic and nucleoplasmic protein concentration, respectively. The overall intra-assay percent coefficient of variation was 9.79% (±6.86) and 9.04% (±7.70), respectively.

### 2.14. Statistical Analysis

Statistical analysis for serum and muscle hormone concentrations, protein metabolite concentrations, and AR-DNA binding activity were performed by utilizing separate 2 × 3 (Condition (LL, HL) × Time (Pre-exercise, 3-h Post-exercise, and 24-h Post-exercise)) factorial analyses of variance (ANOVAs) with repeated measures. Given significant baseline value differences, an ANCOVA was used to with the aforementioned baseline data as a covariate. If a significant interaction was found, simple effects analysis was conducted to determine where the interaction occurred. If a significant interaction was present, analysis of main effects was conducted using the simple effects, pairwise comparisons with a Bonferroni adjustment to compare dependent variables within each independent variable condition. If no interaction was present, then normal pairwise comparisons with a Bonferroni adjustment were used to test main effects. The magnitude of statistical significance was measured by effect size (partial Eta-squared), which estimates the ratio of variance in the dependent variable that is explained by the independent variable. Partial Eta Squared effect sizes (η^2^) are characterized 0.1–0.3 as small, 0.3–0.5 as medium, and ≥0.5 as large [50]. All statistical procedures were performed using SPSS 27.0 software and an alpha level of ≤0.05 was set for all statistical measures.

## 3. Results

### 3.1. Dietary Analysis and Hydration Status

The results of the separate one way repeated measures ANOVAs indicated that there was no significant difference in total calories (F = 1.973, *p* = 0.194, η^2^ = 0.180) or carbohydrate (F = 1.690, *p* = 0.213, η^2^ = 0.158), protein (F = 0.805, *p* = 0.462, η^2^ = 0.082), fat (F = 1.532, *p* = 0.243, η^2^ = 0.145), or fiber intake (F = 0.395, *p* = 0.680, η^2^ = 0.047) between the max testing, LL, or HL conditions. Similarly, total body water did not significantly change (F = 1.521, *p* = 0.285, η^2^ = 0.276) between the max testing, LL, or HL conditions. For packed cell volume, no significant main effect for time (F = 1.257, *p* = 0.299, η^2^ = 0.077) or condition (F = 0.242, *p* = 0.630, η^2^ = 0.016) was observed between conditions.

### 3.2. Resistance Exercise Volume, Rating of Perceived Exertion, and Muscle Soreness

The results of the separate pair samples *t*-tests indicated no significant differences between conditions in the volumes for leg press (*t* = 0.482, *p* = 0.641), barbell bench press (*t* =−0.233, *p* = 0.821), lat pulldown (*t* = 1.297, *p* = 0.227), single leg extension (*t* = −0.860, *p* = 0.412), and total testing session (*t* = −0.482, *p* = 0.641), or RPE (*t* = 1.279, *p* = 0.237). Additionally, no significant main effect for condition (F = 0.813, *p* = 0.380, η^2^ = 0.046) or significant interaction for time and condition (F = 0.396, *p* = 0.676, η^2^ = 0.023) for muscle soreness were observed. However, a significant main effect for time (F = 10.983, *p* < 0.001, η^2^ = 0.392) was observed. Specifically, analysis revealed a significant increase in muscle soreness at 3 h post- (*p* = 0.003) and 24 h post-exercise (*p* = 0.001) compared to pre-exercise.

### 3.3. Serum Total and Free Testosterone Concentration

**Figure 1:**
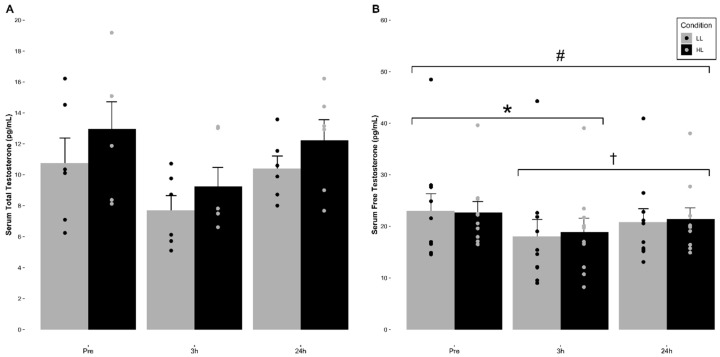
Mean (±SE) and individual changes in serum (**A**) total testosterone and (**B**) free testosterone concentrations in response to volume- and intensity-equated low- and high-load resistance exercise. (**A**): There were no significant changes over time, between conditions, or significant interactions observed (*p* > 0.05). (**B**): * Indicates a significant decrease at 3 h post-exercise compared to pre-exercise values in both conditions (*p* < 0.05). ^#^ Indicates a significant decrease at 24 h post-exercise compared to pre-exercise values in both conditions (*p* < 0.05). ^†^ Indicates 24 h post-exercise was significantly greater than 3 h post-exercise values in both conditions (*p* < 0.05).

The means (±SE) for serum total and free testosterone for each condition are indicated in Figure 1 and Table 2. No significant main effect of condition (F = 4.421, *p* = 0.103, η^2^ = 0.525) or significant time and condition interaction (F = 0.718, *p* = 0.517, η^2^ = 0.152) for serum total testosterone concentrations were observed. However, a significant main effect for time (F = 6.386, *p* = 0.022, η^2^ = 0.615) was observed, which was revealed to be non-significant after adjusting for alpha level inflation via a Bonferroni adjustment (*p* = 0.091).

Regarding serum free testosterone, no significant main effect of condition (F = 0.097, *p* = 0.763, η^2^ = 0.011) or significant time and condition interaction (F = 0.611, *p* = 0.554, η^2^ = 0.064) for serum free testosterone concentrations were observed. However, a significant main effect for time (F = 21.736, *p* < 0.001, η^2^ = 0.707) was observed. Pairwise comparisons revealed that there was a significant decrease in serum free testosterone concentrations at 3 h post-exercise compared to pre-exercise (*p* < 0.001) in both conditions. Additionally, 24 h post-exercise was significantly greater than 3 h post-exercise (*p* = 0.041) but did not return to pre-exercise baseline values (*p* = 0.029) in either condition.

### 3.4. Intramuscular Testosterone and DHT Concentration

**Figure 2:**
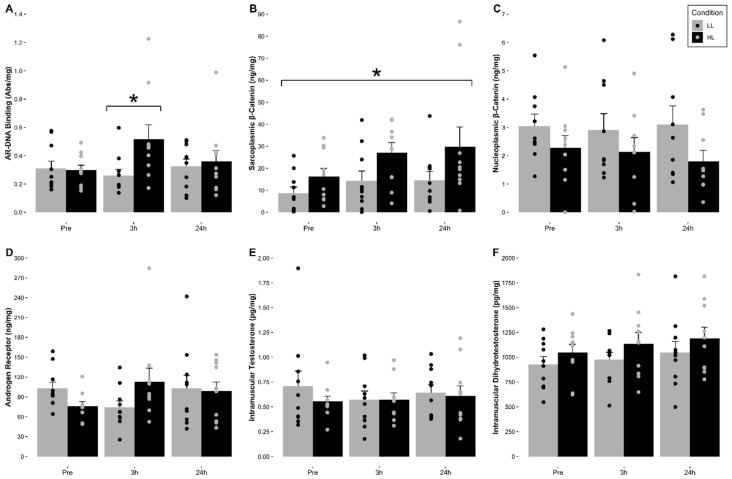
Mean (±SE) and individual changes in (**A**) AR-DNA binding activity, (**B**) sarcoplasmic β-catenin, (**C**) nucleoplasmic β-catenin, (**D**) androgen receptor protein, (**E**) intramuscular testosterone, and (**F**) intramuscular dihydrotestosterone in response to volume- and intensity-equated low- and high-load resistance exercise. (**A**): * Indicates a significant increase in AR-DNA binding activity at 3 h post high-load resistance exercise (*p* < 0.05). (**B**): * Indicates a significant condition effect where high load was significantly greater than low load resistance exercise (*p* < 0.05). (**C**–**F**): There were no significant changes over time, between conditions, or significant interactions observed (*p* > 0.05).

The means (±SE) for intramuscular testosterone and DHT for each condition are indicated in Figure 2 and Table 2. No significant main effect of time (F = 0.451, *p* = 0.644, η^2^ = 0.048), condition (F = 0.643, *p* = 0.443, η^2^ = 0.067), or significant time and condition interaction (F = 0.777, *p* = 0.475, η^2^ = 0.079) for intramuscular testosterone concentrations were observed. For intramuscular DHT, no significant main effect of time (F = 0.350, *p* = 0.711, η^2^ = 0.048), condition (F = 2.680, *p* = 0.146, η^2^ = 0.277), or significant time and condition interaction (F = 0.169, *p* = 0.846, η^2^ = 0.024) for intramuscular DHT concentrations were observed.

### 3.5. Intramuscular Androgen Receptor Protein Content

The mean (±SE) for total intramuscular androgen receptor protein content relative to total muscle protein content (ng/mg) for each condition are indicated in Figure 2 and Table 2. A 2 × 2 (Condition (LL, HL) × Time (3 h Post-, 24 h Post-Exercise)), with pre-exercise as a covariate, factorial ANCOVA with repeated measures was used to account for differences in baseline values. The analysis indicated that no significant main effect for time (F = 0.463, *p* = 0.518, η^2^ = 0.062), condition (F = 2.290, *p* = 0.174, η^2^ = 0.274) or significant time and condition interaction (F = 0.372, *p* = 0.561, η^2^ = 0.030) were observed.

### 3.6. Intramuscular Androgen Receptor-DNA Binding Activity

The mean (±SE) for intramuscular androgen receptor-DNA binding relative to total nucleoplasmic muscle protein content (Abs/mg) for each condition are indicated in Figure 2 and Table 2. No significant main effect of time (F = 1.475, *p* = 0.225, η^2^ = 0.141) or condition (F = 1.406, *p* = 0.266, η^2^ = 0.135) for AR-DNA binding activity were observed. However, a significant interaction for time and condition (F = 4.809, *p* = 0.021, η^2^ = 0.348) was observed. Analysis revealed that there was a significant increase in AR-DNA binding at 3 h post-exercise compared to pre-exercise in the HL condition (*p* = 0.030). 

### 3.7. Intramuscular Sarcoplasmic and Nucleoplasmic β-Catenin Content

The means (±SE) for intramuscular sarcoplasmic and nucleoplasmic β-catenin for each condition are indicated in Figure 2 and Table 2. For the sarcoplasmic fraction, the Mauchly’s test of sphericity indicated that there were violations in sphericity (*p* = 0.002) of the data. Therefore, a Greenhouse–Geisser adjustment was used to meet the needed assumptions to run the appropriate statistical analysis. No significant main effect for time (F = 2.004, *p* = 0.164, η^2^ = 0.182) or significant time and condition interaction (F = 0.504, *p* = 0.513, η^2^ = 0.053) for sarcoplasmic β-catenin content were observed. However, a significant main effect for condition where HL was significantly greater compared to LL when collapsed for time (F = 5.414, *p* = 0.045, η^2^ = 0.376). Regarding nucleoplasmic β-catenin, no significant main effect for time (F = 0.054, *p* = 0.948, η^2^ = 0.011), condition (F = 1.600, *p* = 0.262, η^2^ = 0.242), or significant time and condition interaction (F = 2.474, *p* = 0.134, η^2^ = 0.331) for nucleoplasmic β-catenin content were observed.

## 4. Discussion

This appears to be the first study investigating the effects of RE load in an intensity- and volume-equated manner on AR-DNA binding activity, AR protein content, the influence of the androgenic hormones, as well as both sarcoplasmic and nucleoplasmic β-catenin concentrations on AR signaling. It is generally thought that LL (≤60% 1 RM) and HL (>60% 1 RM) RE have similar outcomes on skeletal muscle hypertrophy when performed to volitional muscular failure [51]. Therefore, the mechanisms which regulate skeletal muscle hypertrophy are also suggested to be identical in both LL and HL scenarios. In contrast to the prevailing theory, our data showed that HL full-body RE significantly increased (~74%) AR-DNA binding at 3 h post-exercise compared to pre-exercise values. Moreover, this was not observed in the LL RE condition across all sampling times which indicates a potential load dependence in AR activation, translocation, and DNA binding. Interestingly, in response to both LL and HL conditions, we observed no significant changes in serum total testosterone, muscle testosterone or DHT concentrations, while serum-free testosterone was significantly decreased at 3 h post- (LL: ~28% vs. HL: ~20%) and 24 h post-exercise (LL: ~11% vs. HL: ~6%) compared to pre-exercise. Similarly, muscle testosterone and DHT concentrations did not significantly change suggesting the decrease/no change in serum-free and total testosterone concentrations did not affect skeletal muscle testosterone and DHT concentrations. Therefore, the observed increase in AR-DNA binding in the HL condition at 3 h post-exercise does not appear to be driven by load-mediated changes in either circulating and/or skeletal muscle androgen concentrations.

For its critical role in facilitating increases in AR signaling, the AR protein response may conceivably be mediating up-regulations in AR-DNA binding activity. However, we observed no significant changes in AR protein content across all time points in either condition. This suggests that our observed increases in AR signaling activity are not driven by changes in AR content. While more research is certainly warranted, this provides further evidence, along with others [13,25,27,28], that AR protein content does not appear to acutely increase in response to RE and provides preliminary evidence that it may not be suggestive of upregulations in AR signaling or predictive of hypertrophic outcomes (AR protein response to chronic resistance training may be more indicative of hypertrophic outcomes; see [52]). Of particular interest, our data demonstrated time-independent, HL-specific greater concentrations of the AR co-activating protein β-catenin relative to LL. Specifically, skeletal muscle sarcoplasmic β-catenin concentrations were ~94% greater in the HL condition versus the LL condition with no significant changes observed in the nucleoplasmic fraction. As a multifunctional protein shown to positively influence a number of processes (cell-cycle progression, cell-to-cell adhesion activity, and ribosome biogenesis) in addition to its ability to co-activate the AR, this load-mediated response provides further evidence of superior anabolic signaling activity in HL compared to LL RE [32,38,53]. Collectively, our findings indicate that, contrary to the current theory of load-mediated RE, AR-DNA binding and β-catenin activity are only increased after HL RE without concomitant increases in serum or muscle androgens or AR content.

The multifunctional Wnt-signaling protein, β-catenin, has been shown to robustly impact stability, activation, and transcriptional activity of the AR [32,54,55]. Theoretically, elevations in this AR co-activating protein directly increase AR activation, translocation, DNA binding, and result in up-regulations in muscle specific gene and eventual protein expression. As previously discussed, our data showed a significant increase in AR-DNA binding activity and greater sarcoplasmic β-catenin content in the absence of significant elevations in serum free and total testosterone, skeletal muscle testosterone and DHT, or AR protein content following HL RE. Considering that nucleoplasmic β-catenin did not appear to be significantly influenced, our data provide ostensible evidence that sarcoplasmic β-catenin may be playing a key regulatory role in encouraging AR–androgen interactions or activating the AR in an androgen-independent manner. Specifically, it appears as though this role is largely mediated through a novel undescribed HL-dependent mechanism that facilitates sarcoplasmic β-catenin accumulation ultimately increasing AR-protein interactions and facilitating AR-DNA binding within the nucleus. This phenomena also ostensibly contradicts the current theory denoting equivocal anabolic-signaling outcomes between HL and LL RE, and provides further evidence Wnt/β-catenin signaling is responsive to mechanical loading [23,36,42]. However, this is the first study to propose this response may be dependent on the resistance or load placed on skeletal muscle.

**Figure 3:**
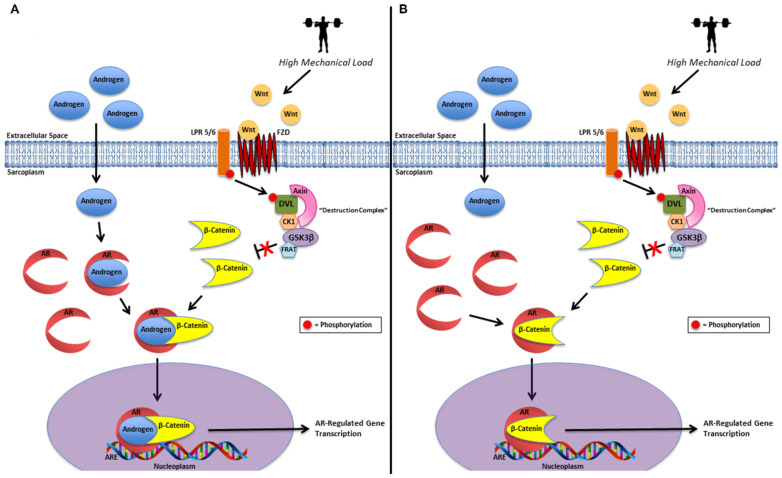
Sarcoplasmic androgen-dependent and androgen-independent AR activation by β-catenin. Two primary hypothetical mechanisms or a combination thereof posited to be associated with our findings: (1) sarcoplasmic stabilization and co-activation of the AR-bound androgen by β-catenin (**A**) and (2) sarcoplasmic androgen-independent activation of the AR by β-catenin (**B**). AR = androgen receptor; LPR 5/6 = low-density lipoprotein receptor-related protein 5 or 6; FZD = frizzled receptor; Wnt = wingless-type mouse mammary tumor virus integration site (Wnt) protein; DVL = disheveled; CK1 = casein kinase 1; GSK3β = glycogen synthase kinase 3β; FRAT = frequently rearranged in advanced T cell lymphomas; ARE = androgen response element).

There is considerable evidence of crosstalk between Wnt/β-catenin and AR signaling in the literature [35,38,39,55,56,57,58]. β-catenin has been reported to activate the AR in both an androgen-dependent [54,55] and androgen-independent [35] manner. Previous in vitro and in vivo animal models offer inconclusive support in determining this potential androgen dependence of β-catenin-AR activity [54,55]. Lack of significant changes in muscle testosterone, DHT, or AR protein content with concomitant elevations in AR-DNA binding activity and greater sarcoplasmic β-catenin content alone does not provide sufficient evidence to make definitive mechanistic inferences about our data. Nevertheless, since we have demonstrated β-catenin’s ability to accumulate in the sarcoplasm, with no changes within the nucleoplasm, we hypothesize our findings may be due to two primary mechanisms or a combination thereof including: (1) sarcoplasmic stabilization and co-activation of the AR-bound androgen by β-catenin and (2) sarcoplasmic androgen-independent activation of the AR by β-catenin (Figure 3) [35,36,54,55,59].

While in vitro and in vivo animal models provide valuable insight into the potential molecular mechanisms governing this response, human models are more comparable given our study design. In a recent study by Spillane et al. [23], they investigated the β-catenin and AR signaling response to an acute bout of lower- and full-body RE. Also of relevance, this study design did not equate RE volume between the two conditions. Therefore, the full-body condition performed significantly more volume than the lower-body condition. Similar to our findings, they found increased AR-DNA binding activity and β-catenin content at 3 h post- and 24 h post-exercise, in addition to elevated serum Wnt4 concentrations at 30 min post-, 1 h post-, and 2 h post-exercise, following the full-body RE bout. Consistent with our observations, no significant changes in serum-free and total testosterone or muscle testosterone and DHT were observed at any time point or condition. However, they did witness a significant increase in AR protein content at 3 h post- and a significant decrease at 24 h post-full body RE. Given that an increase in AR-DNA binding activity occurred at these time points, it further begs the question of whether the AR protein response is facilitating this acute increase in AR signaling activity. Given the incongruent AR protein and AR-DNA binding response in our study and that of Spillane et al. [23], we propose these observed acute elevations in AR signaling are not driven by changes in AR content. Rather, these data indicate up-regulations in Wnt/β-catenin signaling may be facilitating increases in AR-DNA binding which appears reflective of elevations in AR signaling and potentially transcriptional activity. Furthermore, these data collectively suggest this response appears to be sensitive to load as well as the volume of mechanical work placed on skeletal muscle.

Previous research lends support to the notion that differential loading protocols do not dictate hypertrophy or increase AR signaling-related markers in skeletal muscle. A recent 12-week study by Morton et al. [12] concluded that LL (30–50% 1 RM) and HL (75–90% 1 RM) RE performed to volitional failure did not preferentially influence androgen hormone concentrations acutely or dictate skeletal muscle hypertrophy after 12-weeks. Similarly, our data demonstrate systemic and local androgen concentrations may not be significantly impacting AR signaling responses given the observed serum and muscle androgen-independent increase in AR-DNA binding. While the acute nature of our design prevents us from making definitive statements on hypertrophic outcomes and load, our data suggest a potential preferential anabolic response to HL RE as exhibited by the observed load-mediated increase in AR-DNA binding. Due to the overwhelming role volume and intensity play in RE-mediated hypertrophy, we speculate that the study design of Morton et al. [12] was unable to detect a preferential load response or lack thereof due to unequivocal RE volumes performed between HL and LL conditions [45,60]. Specifically, the HL condition only completed ~62% of the total volume completed by the LL condition. By contrast, our design equated both volume and intensity of the different load conditions allowing for the acute effects of load to be carefully disseminated in the context of AR signaling and potential implications in skeletal muscle hypertrophy. Nevertheless, while this study does not demonstrate the effects of varying loads on practical hypertrophic outcomes, it corroborates our data and many others [52,61,62,63,64], providing evidence that systemic hormones are neither related to nor predictive of RE-induced changes in skeletal muscle mass in healthy young male participants.

In RE research, rarely is the effect of load on muscular adaptations examined without being affected by confounding variables such as volume and intensity. Previous research has clearly demonstrated the overwhelming influence volume and intensity have on skeletal muscle hypertrophy and the molecular responses that regulate these adaptations [45,60]. At this point, it appears this is the first study to control for these variables to accurately disseminate the effects of RE load on anabolic signaling pathways suggested to mediate skeletal muscle hypertrophy. The novelty in our study design limits our ability to speculate since no data seem to exist investigating the effects of RE load, in a volume- and intensity-equated manner, on markers of AR signaling. Nevertheless, our findings suggest a preferential load-dependent increase in AR signaling thought to be at least partially mediated through elevations in Wnt/β-catenin-related signaling.

## 5. Conclusions

This appears to be the first study to date investigating the impacts of RE load, in a volume- and intensity-equated manner, on AR-DNA binding activity, serum and muscle androgen concentrations, AR protein content, and sarcoplasmic and nucleoplasmic β-catenin concentrations. In response to HL RE, we observed a significant ~74% increase in AR-DNA binding activity without significant elevations in serum or muscle androgen concentrations or AR protein content. However, sarcoplasmic β-catenin content was ~94% significantly greater when comparing the HL versus LL conditions regardless of time. Collectively, our findings provide evidence that when volume and intensity are equated, the acute AR signaling response to mechanical loading on skeletal muscle appears to be load-mediated. Moreover, the observed up-regulations in AR-DNA binding activity and greater sarcoplasmic β-catenin content suggest a preferential AR signaling response to HL RE. Our data further mechanistically support previous evidence of acute increases in AR signaling not being driven by changes in serum or muscle androgen concentrations nor AR protein content. Rather, AR co-activating proteins, such as β-catenin, may be largely responsible for mediating this response. Unfortunately, we cannot infer causality with the current dataset, considering the (time × condition) interaction effect observed in AR-DNA binding at 3 h was not mirrored by the observed condition-only effect in sarcoplasmic β-catenin. Furthermore, an inability to randomize study conditions to equate volume and intensity and a relatively small sample size are both clear limitations and must be considered when interpreting our findings. Many questions still remain about a variety of factors driving the acute AR signaling response to RE. Better understanding of molecular mechanisms in which AR co-activating proteins, such as β-catenin, interact with the AR may allow for more clarity and provide the necessary context to better explain these occurrences. Future research should investigate the impacts of other AR co-activating proteins and determine their differential impacts on the acute AR signaling response and, by extension, hypertrophic outcomes in skeletal muscle.

## Figures and Tables

**Table 1:** Mean (±standard deviation (SD)) descriptive information of all participants, anthropometrics, health markers, and resistance exercise related variables.

Descriptive	Mean (±SD)
Sample Size	10
Age (year)	23.2 (±4.68)
Height (cm)	176.78 (±0.58)
Total Body Mass (kg)	87.15 (±5.77)
Lean Body Mass (kg)	70.66 (±6.62)
Bone Mineral Content (kg)	2.87 (±0.25)
Fat Mass (kg)	13.62 (±3.54)
Body Fat (%)	15.73 (±4.30)
Resting Heart Rate (bpm)	63.6 (±9.13)
Systolic Blood Pressure (mmHg)	118.2 (±5.77)
Diastolic Blood Pressure (mmHg)	75.4 (±7.66)
Resistance Training Experience (year)	4.68 (±1.85)
Leg Press 1 RM (kg)	464.4 (±93.8)
Barbell Bench Press 1 RM (kg)	116.8 (±12.7)

SD, standard deviation; 1 RM, one-repetition maximum.

**Table 2:** Mean (±SE) of all intramuscular and serum markers of AR—β-Catenin signaling in response to low- (LL) and high-load (HL) resistance exercise.

Condition	Pre-Exercise	3 h Post	24 h Post
AR-DNA Binding (Abs/mg)			
LL	0.31 (±0.05)	0.26 (±0.05)	0.33 (±0.05)
HL	0.30 (±0.04)	0.52 (±0.10) *	0.36 (±0.08)
Sarcoplasmic β-Catenin (ng/mg)			
LL	8.77 (±2.79)	14.38 (±4.38)	14.62 (±3.96)
HL	16.35 (±3.62) *	27.09 (±4.62) *	29.83 (±8.90) *
Nucleoplasmic β-Catenin (ng/mg)			
LL	2.49 (±0.42)	2.28 (±0.58)	3.23 (±0.67)
HL	2.65 (±0.43)	2.46 (±0.50)	1.50 (±0.38)
Androgen Receptor (ng/mg)			
LL	102.85 (±9.46)	74.21 (±9.73)	102.92 (±19.20)
HL	76.20 (±6.55)	113.02 (±20.45)	98.71 (±13.76)
Intramuscular TEST (pg/mg)			
LL	0.71 (±0.15)	0.57 (±0.09)	0.65 (±0.08)
HL	0.55 (±0.05)	0.57 (±0.07)	0.61 (±0.10)
Intramuscular DHT (pg/mg)			
LL	928.32 (±76.59)	975.60 (±72.34)	1047.05 (±113.02)
HL	1048.11 (±81.79)	1137.32 (±111.62)	1190.96 (±111.98)
Serum Total TEST (pg/mL)			
LL	10.75 (±1.62)	7.69 (±0.96)	10.39 (±0.82)
HL	12.95 (±1.76)	9.26 (±1.21)	12.23 (±1.33)
Serum Free TEST (pg/mL)			
LL	23.03 (±3.25)	18.05 (±3.28) *	20.84 (±2.58) *
HL	22.67 (±2.13)	18.86 (±2.73) *	21.41 (±2.18) *

Data were analyzed using separate 2 × 3 factorial analyses of variance (ANOVAs) with repeated measures and an alpha level of ≤0.05. Protein markers are expressed relative to total protein content of the appropriate cell compartment or total cellular protein content. LL, low load; HL, high load; AR, androgen receptor; TEST, testosterone; DHT, dihydrotestosterone. * Indicates a significant main effect for time, condition, or time and condition interaction (*p* < 0.05).
